# Real-Time Cardiac Arrhythmia Classification Using TinyML on Ultra-Low-Cost Microcontrollers: A Feasibility Study for Resource-Constrained Environments

**DOI:** 10.3390/bioengineering13050532

**Published:** 2026-05-01

**Authors:** Misael Zambrano-de la Torre, Sebastian Guzman-Alfaro, Andrea Acuña-Correa, Manuel A. Soto-Murillo, Maximiliano Guzmán-Fernández, Ricardo Robles-Ortiz, Karen E. Villagrana-Bañuelos, Jose G. Arceo-Olague, Carlos H. Espino-Salinas, Ana G. Sánchez-Reyna, Erik O. Cuevas-Rodriguez

**Affiliations:** 1 Unidad Académica de Ingeniería Eléctrica, Universidad Autónoma de Zacatecas, Zacatecas 98160, Mexicoalejandro.somu@uaz.edu.mx (M.A.S.-M.); arceojg@uaz.edu.mx (J.G.A.-O.);; 2 Unidad Académica de Medicina Humana, Universidad Autónoma de Zacatecas, Jardín Juárez 147, Centro, Zacatecas 98000, Mexico

**Keywords:** TinyML, ECG, arrhythmia classification, embedded systems, edge AI, low-cost healthcare

## Abstract

Recent advances in edge computing and Tiny Machine Learning (TinyML) have enabled the deployment of artificial intelligence models directly on microcontrollers with extremely limited computational and memory resources. In this context, this work presents the design, implementation, and validation of a real-time cardiac arrhythmia classification system based on a quantized one-dimensional convolutional neural network (1D-CNN), deployed on an 8-bit Arduino UNO microcontroller. The proposed system integrates end-to-end processing, including ECG signal acquisition using a low-cost AD8232 analog front-end, signal preprocessing, heartbeat segmentation, classification, and real-time visualization on an OLED display. The model was trained and evaluated using the MIT-BIH Arrhythmia Database, considering a reduced three-class problem (Normal, Ventricular, and Supraventricular) to meet the constraints of ultra-low-cost hardware deployment. Under benchmark conditions, the quantized model achieved an accuracy of 97.6%, with a memory footprint below 24 KB and an average inference time of 200 ms per heartbeat, enabling real-time operation on a resource-constrained microcontroller. Real-time experiments were conducted using signals acquired from healthy volunteers to validate system functionality, although no annotated ground truth was available for these recordings, and therefore no diagnostic performance was derived from them. The results demonstrate the feasibility of deploying lightweight deep learning models on ultra-constrained embedded systems using the TinyML paradigm, implemented using TensorFlow 2.15 and TensorFlow Lite. This work should be interpreted as a proof-of-concept platform that highlights the trade-off between classification performance and hardware limitations, providing a foundation for future development of low-cost cardiac monitoring technologies in resource-limited environments.

## 1. Introduction

Cardiovascular diseases remain the leading cause of mortality both in Mexico and worldwide, according to reports from the World Health Organization (WHO) and the National Institute of Statistics and Geography (INEGI). This substantial global health burden arises from a combination of multiple factors, among which limited access to specialized medical care in rural and marginalized regions of Mexico is particularly significant. These constraints reflect deep-rooted structural inequalities within public healthcare systems, hindering early detection and timely management of cardiovascular conditions in vulnerable populations [1,2,3,4].

In the Mexican context, recent studies have also explored the use of machine learning techniques for cardiovascular disease classification, highlighting the growing interest in data-driven approaches to support diagnosis and screening in the country [5].

The proper functioning of the heart directly influences individuals’ quality of life; therefore, the ability to examine, monitor, and accurately analyze cardiac activity has become a priority from both medical and technological perspectives [6,7,8]. The human body represents a continuous source of physiological information, constantly generating biological signals that reflect its internal and functional state [9,10]. In particular, the heart conveys its condition through electrical and mechanical signals whose interpretation enables the detection of structural and functional abnormalities. Over recent years, numerous analytical and mathematical modeling approaches have been developed to extract meaningful information from these signals, including time–frequency analysis methods, wavelet decomposition, cepstral coefficients, and both traditional and deep learning-based classifiers. Recent studies have shown that hybrid representations—such as the combination of Mel-Frequency Cepstral Coefficients (MFCCs) and wavelet-derived features coupled with robust classifiers like support vector machines or convolutional neural networks—can achieve competitive and even state-of-the-art performance in cardiac signal classification tasks. Nevertheless, these approaches often rely on computationally complex architectures, large-scale datasets, extensive validation procedures, and advanced hardware resources, which substantially limits their practical deployment in infrastructure-constrained environments [11,12,13,14,15]. Consequently, despite their high accuracy under controlled conditions, many existing models remain impractical for use in rural clinics or remote regions, where energy consumption, hardware cost, and connectivity requirements pose significant barriers. More recently, advances in artificial intelligence have driven the adoption of computer-aided diagnosis (CAD) systems, highlighting the need for alternative solutions that balance robustness, computational efficiency, and deployment feasibility in low-resource healthcare settings [16].

Among existing tools for cardiac evaluation, such as the stethoscope, echocardiography, computed tomography, and other imaging-based modalities [17,18,19], the electrocardiograph (ECG) remains the clinical standard for detecting abnormalities in cardiac rhythm and morphology. ECG-based analysis enables the identification of a broad spectrum of arrhythmias and other pathological cardiac conditions, making it an essential instrument in both preventive and emergency medicine [20,21,22]. Over the past few decades, significant advances have been made in ECG signal acquisition, portability, and monitoring capabilities. In parallel, a growing number of portable and low-cost ECG systems have emerged for home-based or non-specialist use, including wearable devices, smartwatch-integrated ECG modules, smartphone-connected ECG adapters, and compact portable monitoring platforms. Some of these solutions are marketed as certified medical devices, while others function as consumer-oriented monitoring tools with limited diagnostic scope. However, the integration of automated diagnostic interpretation into low-cost ECG systems has progressed more slowly. In many cases, commercially available ECG devices still rely on proprietary software, centralized processing, or specialist interpretation. Furthermore, mid-range clinical ECG instrumentation can cost around USD 1000 or more depending on the technical specifications, while fully certified medical-grade systems may be substantially more expensive due to regulatory compliance, safety validation, and certification requirements rather than hardware cost alone [23]. These factors constrain the widespread deployment of advanced ECG-based diagnostic systems in rural or low-resource healthcare settings. These systems are typically implemented on a wide range of hardware platforms, including ARM-based microcontrollers, system-on-chip (SoC) devices, and embedded Linux platforms such as Raspberry Pi, which provide higher computational capabilities compared to ultra-low-cost 8-bit microcontrollers.

In this context, the integration of artificial intelligence into low-cost medical instrumentation represents a transformative opportunity for public health. AI-driven systems can enhance the interpretability of ECG signals, enabling the automated identification of cardiac arrhythmias and other abnormalities without requiring continuous specialist supervision. This integration directly addresses two critical limitations of the Mexican healthcare system: the shortage of trained cardiology professionals and the unequal distribution of diagnostic equipment [24,25]. By leveraging intelligent embedded systems, it becomes feasible to extend reliable and accessible cardiac screening and decision-support tools to remote and underserved communities, thereby supporting early cardiac assessment and contributing to a reduction in preventable mortality.

Recent advances in edge computing and *Tiny Machine Learning* (TinyML) have further expanded this possibility by enabling the deployment of neural networks directly on microcontrollers with extremely limited computational and memory resources. For example, Farag (2023) [26] reported an ECG-based arrhythmia classification model capable of discriminating five types of cardiac arrhythmias, achieving an overall classification accuracy of 98.18% on a standard benchmark dataset.

Recent developments have also explored portable and spectrogram-based deep learning systems for ECG analysis, further emphasizing the need for solutions that balance accuracy and real-world deployability [27,28]. These technologies allow real-time inference on-device, eliminating the need for external servers or constant network connectivity—an essential requirement for isolated clinical environments.

### Main Contributions

The main contributions of this work are summarized as follows:

1. Real-time TinyML deployment on ultra-constrained hardware: Demonstration of real-time arrhythmia classification using a compact and quantized 1D-CNN deployed on an 8-bit microcontroller with extremely limited memory and computational resources.
2. End-to-end standalone embedded integration: Full integration of ECG signal acquisition, preprocessing, segmentation, classification, and visualization into a fully standalone embedded system.
3. Operation without external computing infrastructure: Implementation of a TinyML-based solution that operates without external processing units, cloud connectivity, or floating-point hardware.
4. Analysis of the performance–deployability trade-off: Evaluation of the trade-off between classification performance and deployability under severe hardware constraints, highlighting the feasibility limits of ultra-low-cost medical support systems.

Therefore, the objective of this work is to develop and validate a real-time cardiac arrhythmia classification system based on TinyML, implemented using TensorFlow 2.15 and TensorFlow Lite on ultra-low-cost embedded hardware. The proposed system employs a quantized one-dimensional convolutional neural network (1D-CNN) trained on the MIT-BIH Arrhythmia Database and deployed on an 8-bit Arduino microcontroller. Rather than introducing a novel deep learning architecture, this work focuses on demonstrating the technical feasibility of compressing, integrating, and executing an arrhythmia classifier under severe hardware constraints. In this sense, the contribution of this study is engineering-oriented rather than algorithmic, emphasizing deployment feasibility within the TinyML paradigm over architectural innovation. The proposed platform should be interpreted as a proof-of-concept embedded system rather than as a certified medical device, providing a practical step toward accessible and decentralized cardiac screening tools for rural clinics in Mexico and similar resource-limited contexts.

It is important to emphasize that the proposed system is not intended as a replacement for clinical-grade ECG devices, but rather as a low-cost screening and decision-support tool. The main contribution lies in demonstrating the feasibility of deploying arrhythmia classification models under extreme hardware constraints, enabling potential applications in educational settings and in resource-limited healthcare environments.

## 2. Materials and Methods

**Figure 1:**
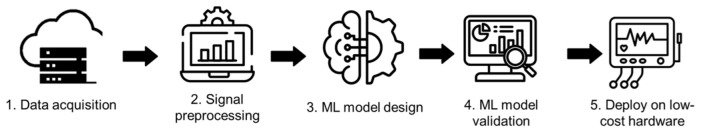
General workflow of the proposed methodology for real-time arrhythmia classification using TinyML.

The development of the proposed system for real-time cardiac arrhythmia classification using TinyML was structured into five main stages: (1) data acquisition, (2) signal preprocessing, (3) machine learning model design, (4) model validation, and (5) implementation on low-cost embedded hardware. Figure 1 schematically illustrates the overall workflow of the methodology employed in this study.

### 2.1. Data Acquisition

For the training and quantitative validation of the proposed model, the MIT-BIH Arrhythmia Database, publicly available through PhysioNet [29,30], was used. This dataset represents the de facto benchmark for arrhythmia classification research, as it has been extensively validated and employed in numerous studies on automated detection of cardiac events since its creation in 1980. All statistical performance metrics reported in this study, including accuracy, precision, recall, F1-score, and AUC, were obtained exclusively from this public benchmark dataset.

The dataset comprises 48 ambulatory electrocardiogram (ECG) recordings collected from 47 subjects (25 men and 22 women), aged between 23 and 89 years. Each record has a duration of 30 min and was acquired using two simultaneous leads, digitized at a sampling frequency of 360 Hz, with an 11-bit resolution and an amplitude range of ±5 mV.

In this study, only the Lead II signal was used, as it provides the most stable and morphologically representative view of the QRS complex in single-channel configurations, making it particularly suitable for low-cost portable systems. Each ECG record contains beat-by-beat annotations reviewed by expert cardiologists, enabling accurate labeling of heartbeat types. In total, the database includes more than 110,000 annotated heartbeat segments, which serve as independent samples for model training and evaluation in this study. These recordings were originally categorized according to the Association for the Advancement of Medical Instrumentation (AAMI) standard.

Although the MIT-BIH database follows the AAMI standard, which defines five heartbeat categories (Normal (N), Supraventricular (SV), Ventricular (V), Fusion (F), and Unknown (Q)), this study focuses on a reduced subset consisting of three classes: Normal (N), Ventricular (V), and Supraventricular (S). This reduction was intentionally adopted to ensure reliable deployment on resource-constrained hardware, particularly the Arduino UNO platform, where memory and computational limitations restrict the complexity of the classification task.

It is important to note that this simplification reduces the clinical granularity of the system, since other relevant heartbeat categories included in the AAMI framework are not considered in the present implementation. Therefore, the proposed classifier should be interpreted as a proof-of-concept screening-oriented system rather than as a comprehensive arrhythmia classification solution.

For model training, the dataset was divided into three stratified subsets: 60% for training, 20% for validation, and 20% for testing. The partitioning was performed at the heartbeat level (beat-wise), ensuring that each subset maintained the original class proportions and preventing bias toward the predominance of normal beats. However, this strategy does not enforce inter-patient separation, meaning that heartbeat segments from the same subject may appear in both training and testing subsets. As a result, data leakage may occur, and the reported performance can be optimistically biased due to shared patient-specific morphological patterns across subsets.

Therefore, the present evaluation should not be interpreted as a strict patient-independent validation. Instead, it should be understood as a benchmark-level technical evaluation intended to assess whether a lightweight arrhythmia classifier can be trained, compressed, and deployed under severe embedded hardware constraints. A more rigorous inter-patient assessment using patient-wise partitioning remains necessary for clinically realistic validation and is part of future work.

Thus, the testing subset contains heartbeat segments rather than patient cases, and each segment corresponds to an ECG window of 250 samples (approximately 0.7 s).

It is important to distinguish between the two validation levels used in this work: the benchmark-based quantitative evaluation of the classification model was performed exclusively on the MIT-BIH dataset, whereas the prototype-level real-time experiments were conducted separately to verify signal acquisition and embedded inference feasibility.

No external dataset, annotated private dataset, or real-world low-cost noisy ECG dataset was used for quantitative model validation in this study. Therefore, the reported performance metrics should be interpreted as benchmark-based results and not as direct evidence of generalization to practical deployment conditions.

### 2.2. Signal Preprocessing

Before the training and deployment of the classification model, digital signal processing (DSP) techniques were applied to ensure the quality of the ECG signals and to remove common artifacts associated with motion, respiration, and power line interference. The objective of this stage was to improve the signal-to-noise ratio (SNR) and normalize the input segments used by the machine learning model. The preprocessing workflow consisted of three main steps: filtering, normalization, and segmentation.

#### 2.2.1. Filtering

The raw ECG signals from the MIT-BIH dataset were processed using a fourth-order digital Butterworth band-pass filter with cutoff frequencies of 0.5 Hz and 40 Hz. The lower cutoff frequency (0.5 Hz) was selected to remove baseline wander, which is typically caused by respiration or electrode movement. The upper cutoff frequency (40 Hz) was used to attenuate high-frequency interferences, mainly due to muscle activity and power line noise.

Although the standard ECG bandwidth can extend from approximately 0.05 Hz to 100 Hz in clinical-grade acquisition systems, the selected range (0.5–40 Hz) was intentionally adopted as a practical trade-off for embedded implementation. This frequency band preserves the most relevant morphological components of the ECG signal, including the P wave, QRS complex, and T wave, which are sufficient for heartbeat-level classification tasks. At the same time, it reduces noise and computational complexity, which is critical for real-time processing on resource-constrained hardware such as 8-bit microcontrollers.

Filtering was implemented in the time domain using a zero-phase forward–backward approach to preserve waveform morphology and avoid phase distortion of the QRS complex, whose integrity is critical for heartbeat classification.

#### 2.2.2. Normalization

After filtering, each signal was scaled using Min–Max normalization according to the following equation:

(1) $$x_{norm}\left(t\right)=\frac{x\left(t\right)-x_{min}}{x_{max}-x_{min}}$$

where x(t) represents the amplitude of the ECG signal at time *t*, and $x_{norm}\left(t\right)$ is the normalized signal within the range [0, 1]. The parameters $x_{min}$ and $x_{max}$ correspond to the minimum and maximum amplitude values computed within each individual heartbeat segment, respectively.

This segment-wise normalization ensures that each input sample is independently scaled, reducing inter-signal variability and improving model convergence while preserving the relative morphology of each heartbeat.

This approach is particularly suitable for embedded applications, where signal amplitude variability may arise from sensor characteristics, electrode placement, and environmental noise.

#### 2.2.3. Segmentation

With the filtered and normalized signals, R-peak detection was performed to segment the continuous ECG signal into windows centered on each heartbeat. An adapted version of the Pan–Tompkins algorithm was implemented, which is widely used for real-time QRS complex detection. The process involves: (1) differentiation to emphasize steep slopes, (2) squaring of the signal, (3) moving window integration, and (4) application of an adaptive threshold to identify R-peaks.

Each heartbeat window was extracted by centering around the detected R-peak and extending 125 samples before and 125 samples after it (a total of 250 samples), which corresponds approximately to 0.7 s at a sampling frequency of 360 Hz. This window size ensures the inclusion of the complete QRS complex as well as the adjacent P and T waves, thus preserving the morphological integrity of each beat.

Accordingly, each input sample to the model corresponds to a single heartbeat-centered ECG segment rather than to a full patient record. The final result of this process was a set of standardized, labeled heartbeat segments (according to the selected three-class scheme), which were used as direct input to the 1D CNN model.

### 2.3. ML Model Design

The proposed model was based on a lightweight one-dimensional convolutional neural network (1D-CNN), specifically selected to operate on microcontrollers with limited computational resources. The choice of a compact and well-established 1D-CNN architecture was intentional, as the purpose of this study was not to maximize architectural complexity, but to ensure a suitable balance between classification performance and deployability on ultra-constrained embedded hardware.

1D-CNNs are particularly effective for analyzing temporal biomedical signals such as ECG [31,32,33], as they can learn hierarchical representations of heartbeat morphology without requiring manual feature extraction. This type of architecture has been widely used in the literature for arrhythmia classification tasks and has demonstrated strong performance across multiple benchmark datasets [34,35].

In addition, recent literature has extensively explored artificial intelligence techniques for ECG signal processing, including deep learning-based classification, IoT-integrated monitoring systems, and low-power hardware implementations [36,37,38,39,40]. Comprehensive reviews highlight the rapid evolution of AI-driven arrhythmia detection methods and the importance of preprocessing strategies for improving classification robustness [41,42].

Furthermore, several recent studies have proposed real-time and embedded ECG classification systems based on deep neural networks and hardware-efficient implementations, demonstrating the growing interest in deployable solutions for cardiac monitoring [43,44,45].

It is important to emphasize that the objective of this work is not to introduce a novel neural network architecture, loss function, or training strategy. Instead, the focus is on evaluating the feasibility of deploying a compact and well-established model under severe embedded constraints using the TinyML paradigm. Therefore, the methodological contribution of this study lies in the adaptation, optimization, and integration of the model into an ultra-constrained hardware environment.

#### 2.3.1. Model Architecture

The final model architecture is shown in Table 1 and consists of the following layers: (1) two one-dimensional convolutional layers with ReLU activation functions, (2) max pooling layers for dimensionality reduction, and (3) a flatten layer followed by two fully connected dense layers, with the final layer employing a Softmax activation function for multiclass classification.

The model contains approximately 18,500 trainable parameters, providing a suitable balance between representational capacity and computational efficiency. This configuration was carefully selected to ensure that the model could be converted and executed within the memory constraints of the Arduino UNO (Arduino, Ivrea, Italy), (32 KB Flash, 2 KB SRAM) once quantized to 8-bit integer precision.

#### 2.3.2. Training Process

Model training was carried out using the TensorFlow 2.15 framework (Python 3.10) on a local environment equipped with a GPU. The loss function used was categorical cross-entropy, suitable for multiclass classification tasks, and the optimizer was Adam, with the hyperparameters summarized in Table 2.

The segmented ECG signals were fed into the model as matrices of size (250, 1), corresponding to 250 samples per beat and a single channel. Batch normalization was applied to accelerate convergence, and a dropout rate of 20% was used to reduce overfitting. Weights were initialized using the uniform method, and model accuracy was monitored on the validation set throughout training.

The model was trained to classify heartbeats into three categories: Normal (N), Ventricular (V), and Supraventricular (S).

Although the study does not operate at the patient level, the heartbeat-level formulation provides a large number of annotated input samples derived from the MIT-BIH Arrhythmia Database, which is sufficient for training and validating compact CNN-based classifiers under benchmark conditions.

#### 2.3.3. Optimization for Deployment (TinyML)

After training, the model was converted to the TensorFlow Lite for Microcontrollers (TFLM) format using the following conversion command:

tflite_convert --optimize=OPTIMIZE_FOR_SIZE --output_file=model_int8.tflite

A post-training quantization was then applied, converting floating-point weights to 8-bit integers (INT8). This process reduced the model size by approximately 75% while maintaining accuracy within 1% of the original floating-point version. The resulting quantized model was converted to a C array file (model.h) using the xxd tool, allowing direct integration into the Arduino UNO firmware.

The quantized model achieved a final size of less than 22 KB, enabling complete execution on the microcontroller without requiring a floating-point unit (FPU). The average inference time measured was approximately 200 ms per heartbeat. This value corresponds exclusively to the execution of the quantized 1D-CNN model on the microcontroller. Signal preprocessing steps, including filtering and R-peak detection, are performed separately and introduce additional computational overhead. Nevertheless, the inference time alone satisfies real-time processing requirements for beat-by-beat arrhythmia detection.

### 2.4. ML Model Validation

The performance of the proposed model was evaluated using a stratified five-fold cross-validation scheme to estimate classifier performance under benchmark conditions. The unit of analysis in this evaluation was the heartbeat segment rather than the patient record. In other words, each sample used for training, validation, and testing corresponds to a segmented heartbeat of 250 samples (approximately 0.7 s). It is important to note that the cross-validation procedure was conducted at the heartbeat level (beat-wise), without enforcing inter-patient separation. Consequently, heartbeat segments from the same patient may appear across different folds.

This evaluation strategy can inflate performance estimates, since the model may partially benefit from patient-specific morphological patterns already seen during training. For this reason, the reported metrics should be interpreted as benchmark-level technical results rather than as a strict measure of generalization to unseen patients. A patient-wise validation protocol is required to assess clinically realistic inter-patient performance and is planned for future work. In each iteration, the dataset was divided into five balanced subsets, maintaining the original class proportions according to the selected three-class scheme.

Four folds were used for training and one for testing, repeating the process until each subset served once as the validation set. The mean values of the evaluation metrics obtained from the five folds were reported as the final performance indicators.

#### Performance Metrics

Since the problem corresponds to a supervised multiclass classification task, the evaluation followed the metrics recommended by the Association for the Advancement of Medical Instrumentation (AAMI) and the IEEE EMBS Standards Committee for arrhythmia classifier assessment. The selected metrics were Accuracy, Precision, Recall (Sensitivity), F1-score, and the Area Under the ROC Curve (AUC). These metrics were computed from the confusion matrix obtained for each fold using the following equations:

(2) $$\mathrm{Accuracy}=\frac{TP+TN}{TP+TN+FP+FN}$$

(3) $$\mathrm{Precision}=\frac{TP}{TP+FP}$$

(4) $$\mathrm{Recall}=\frac{TP}{TP+FN}$$

(5) $$F_{1}=2\times \frac{\mathrm{Precision}\times \mathrm{Recall}}{\mathrm{Precision}+\mathrm{Recall}}$$

where: TP (True Positives): number of correctly classified instances belonging to the positive class; TN (True Negatives): number of correctly classified instances not belonging to the positive class; FP (False Positives): number of instances incorrectly classified as positive; and FN (False Negatives): number of positive instances incorrectly classified as negative.

For the multiclass case, the metrics were computed using a weighted average, accounting for the number of instances in each class, as expressed in Equation (6):

(6) $$M_{weighted}=\frac{\sum_{i=1}^{C}n_{i}\times M_{i}}{\sum_{i=1}^{C}n_{i}}$$

where $M_{i}$ represents the individual metric for class *i*, $n_{i}$ the number of samples in that class, and *C* the total number of classes (in this study, C=3).

This evaluation approach ensured that the reported results reflected the classifier’s generalization capability while mitigating overfitting and class imbalance effects.

### 2.5. Deployment on Low-Cost Hardware

To validate the implementation on low-cost embedded hardware, the optimized model was deployed on an Arduino UNO (ATmega328P) platform, selected for its affordability, low power consumption, and extensive support within open-source development environments. This stage demonstrated the feasibility of running a real-time arrhythmia classifier under the TinyML paradigm, using a conventional 8-bit microcontroller.

It is important to emphasize that the proposed prototype does not include medical-grade electrical isolation and was developed exclusively for research and educational purposes. All experiments involving signal acquisition were conducted under controlled laboratory conditions using battery-powered configurations to avoid direct connection to mains-powered systems.

Therefore, the system should not be considered safe for clinical or unsupervised use. A compliant medical device would require proper electrical isolation (e.g., isolation amplifiers) and adherence to international safety standards such as IEC 60601. The present implementation should be interpreted strictly as a proof-of-concept platform.

The complete system consists of three main modules:

1. *ECG Signal Acquisition:* The AD8232 (Analog Devices, Norwood, MA, USA) analog front-end module was used to acquire and condition the ECG signal through amplification and band-pass filtering (0.5–40 Hz). The AD8232 was selected due to its low cost, compact design, and high level of integration, which makes it particularly suitable for rapid prototyping and deployment in resource-constrained environments. This integrated analog front-end includes amplification and basic filtering stages, simplifying hardware implementation compared to more complex solutions.
  Although more advanced analog front-end systems (e.g., multi-channel or higher-resolution devices) are available, they typically involve higher cost, increased design complexity, and greater power consumption. In contrast, the AD8232 provides a practical trade-off between functionality and accessibility for proof-of-concept systems based on the TinyML paradigm.
  The analog output of the AD8232 was connected directly to pin A0 of the Arduino UNO, allowing signal digitization via its 10-bit ADC at a sampling frequency of approximately 360 Hz, thus emulating the conditions of the MIT-BIH dataset.
2. *Local Processing and Inference (TinyML):* The pre-trained and quantized (8-bit) 1D-CNN model was converted to TensorFlow Lite for Microcontrollers (TFLM) format and integrated into the Arduino firmware as a C array file (model.h). Given that the UNO has only 32 KB of Flash memory and 2 KB of SRAM, several optimizations were implemented: (a) reduction in the number of filters and layers, (b) full integer quantization (int8), and (c) use of static buffers for inference. The average inference time per heartbeat window remained below 200 ms, enabling real-time classification of each detected beat.
3. *Visual Interface and Communication:* The classification results were displayed on a 0.96-inch OLED screen based on the SSD1306 controller, connected via the $I^{2}$C bus (A4–A5). The display shows the detected heartbeat type (e.g., Normal, Ventricular Premature, Supraventricular) along with an estimated heart rate indicator.

The electronic components that make up the system and their interconnections are summarized in Table 3.

**Figure 2:**
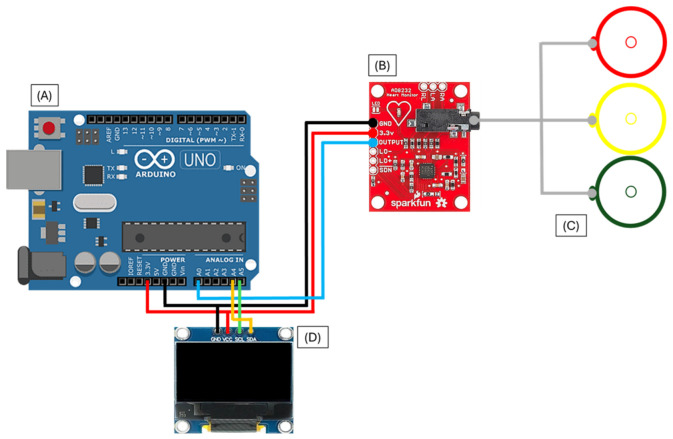
Connection diagram of the electronic system. (**A**) Arduino UNO, (**B**) AD8232 ECG module, (**C**) electrodes, and (**D**) 0.96" OLED display.

Figure 2 illustrates the connection diagram of the system, showing the wiring between the Arduino UNO, AD8232 module, and OLED display.

**Figure 3:**
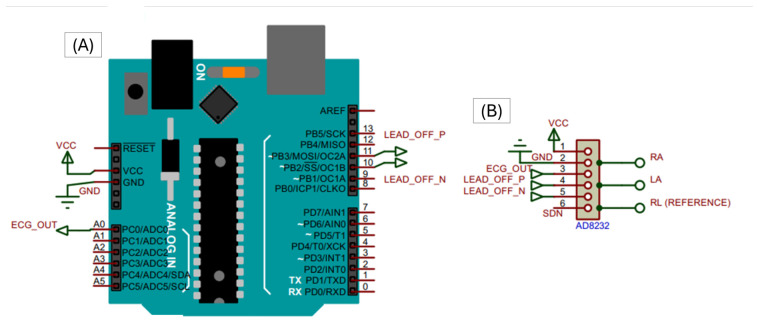
Electrical schematic of the ECG acquisition system based on the AD8232 analog front-end (**B**) and its interface with the Arduino UNO (**A**). The labels A0–A5 correspond to the analog input pins of the Arduino UNO, while 0–13 indicate the digital I/O pins. Pins 1–6 on the AD8232 represent the module connections (GND, VCC, OUTPUT, LO+, LO–, SDN). The signals ECG_OUT, LO+ and LO– correspond to the analog output and lead-off detection signals. RA, LA, and RL denote the right arm, left arm, and reference electrodes, respectively.

Additionally, Figure 3 presents the electrical schematic of the ECG acquisition stage, including the AD8232 analog front-end and its interface with the microcontroller.

The diagram represents the conceptual interconnection of the system components for prototype implementation purposes and does not correspond to a certified medical-grade electrical design.

The schematic highlights the analog signal acquisition path and provides a clearer representation of the hardware-level design, complementing the high-level connection diagram shown previously.

This deployment demonstrates that a basic TinyML diagnostic system can be implemented on ultra-low-cost hardware (below 25 USD), maintaining autonomous and portable operation suitable for use in rural or resource-limited environments. The use of an 8-bit microcontroller validates the minimal feasibility of TinyML in educational and rural contexts, proving that local inference is possible without dependence on ARM processors or continuous connectivity. This stage of the study focuses on technological and functional validation of the embedded system rather than on clinical diagnosis. Accordingly, the hardware implementation should be interpreted as a proof-of-concept platform, while the quantitative classification metrics remain those obtained from the benchmark dataset described in Section 2.1.

## 3. Results and Discussion

This section presents and discusses the experimental results obtained from the implementation of the proposed real-time arrhythmia classification system based on TinyML.

### 3.1. Training and Validation Results

**Figure 4:**
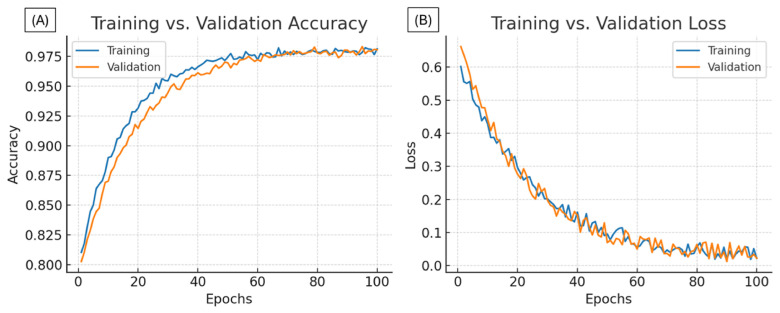
Evolution of (**A**) training and validation accuracy, and (**B**) training and validation loss over 100 epochs. The validation accuracy stabilized after epoch 40, reaching 98.3%, while the validation loss remained below 0.08, indicating strong generalization.

The proposed one-dimensional convolutional neural network (1D-CNN) was trained using heartbeat segments extracted from the MIT-BIH Arrhythmia Database, following the procedure described in the Materials and Methods section. During training, a stable convergence was observed for both the training and validation datasets, with no evidence of significant overfitting, as shown in Figure 4.

A stratified five-fold cross-validation scheme was applied to obtain a robust estimation of the model’s general performance. The average evaluation metrics obtained are summarized in Table 4.

The high F1-Score (97.76%) reflects a robust harmonic balance between precision and sensitivity, indicating that the model minimizes the trade-off between false positives and false negatives. From a clinical perspective, the Recall (Sensitivity) of 97.60% is the most critical metric for a screening device, as it quantifies the system’s ability to correctly identify pathological events. A high sensitivity significantly reduces the risk of ‘missed diagnoses’ (Type II errors), which are particularly dangerous in cardiac monitoring. The consistency of these results across the five folds confirms that the model relies on stable morphological features rather than memorizing artifacts specific to a subset of data.

**Figure 5:**
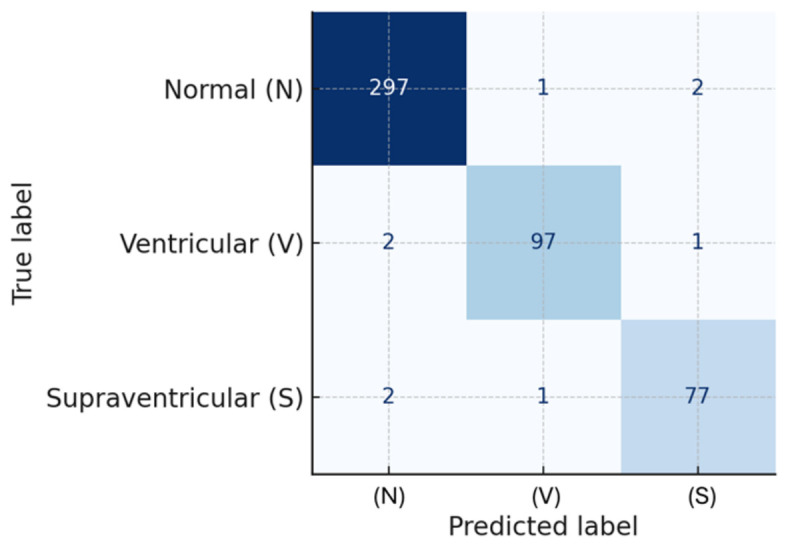
Normalized confusion matrix obtained from the heartbeat-segment-level evaluation for the three classes: Normal (N), Ventricular (V), and Supraventricular (S).

It should also be noted that all reported metrics were computed at the heartbeat-segment level. Therefore, the samples represented in the evaluation correspond to segmented ECG beats of approximately 0.7 s each, rather than complete patient recordings. The classification performance across the three classes is further illustrated by the normalized confusion matrix shown in Figure 5.

The model achieved class-wise recall values of 99.1% for N beats, 97.4% for V beats, and 96.5% for S beats. From a clinical perspective, the recall (sensitivity) values for the pathological classes—Ventricular (97.4%) and Supraventricular (96.5%)—are particularly relevant, as they reflect the system’s ability to correctly identify abnormal cardiac events. In this context, false negatives (i.e., missed abnormal beats) represent the most critical type of error, since they may lead to undetected arrhythmias and delayed medical attention.

Conversely, false positives (i.e., normal beats incorrectly classified as abnormal) are less critical from a safety standpoint, although they may result in unnecessary alerts or additional clinical evaluations. Therefore, the high sensitivity observed across all classes suggests that the proposed system is suitable for screening-oriented applications, where minimizing missed detections is a priority. Although specificity was not explicitly reported for each class, it can be inferred from the confusion matrix. A more detailed class-wise specificity analysis and risk-based evaluation of misclassification errors are considered as part of future work for clinical validation.

These results position the model within the performance range of more complex deep learning architectures such as CNN–LSTM or ResNet, while significantly reducing the number of parameters (≈18,500) and operating without floating-point units. This balance between precision and computational efficiency represents a fundamental step toward its deployment under the TinyML paradigm, where memory and processing limitations are critical factors.

Furthermore, the smooth training behavior (absence of abrupt oscillations in loss curves) confirms the effectiveness of the regularization techniques used, particularly batch normalization and a dropout rate of 20%, which reduced variance without compromising learning capacity.

Overall, the findings indicate that, the 1D-CNN achieved competitive and consistent performance across all folds, demonstrating its ability to capture relevant morphological patterns in ECG signals with a compact parameter set suitable for deployment on low-cost microcontrollers.

### 3.2. Model Quantization and Optimization Impact

To enable the deployment of the proposed classifier on low-cost embedded hardware, the trained model was converted to the TensorFlow Lite for Microcontrollers (TFLM) format. A post-training quantization strategy was applied, transforming 32-bit floating-point weights into 8-bit integers (INT8). This process substantially reduced the memory footprint of the network without significantly compromising its predictive accuracy.

The quantization resulted in a 75% reduction in model size, allowing the entire 1D-CNN to fit within the 32 KB Flash memory available on the Arduino UNO (ATmega328P). Table 5 summarizes the main performance metrics of the model before and after quantization.

**Figure 6:**
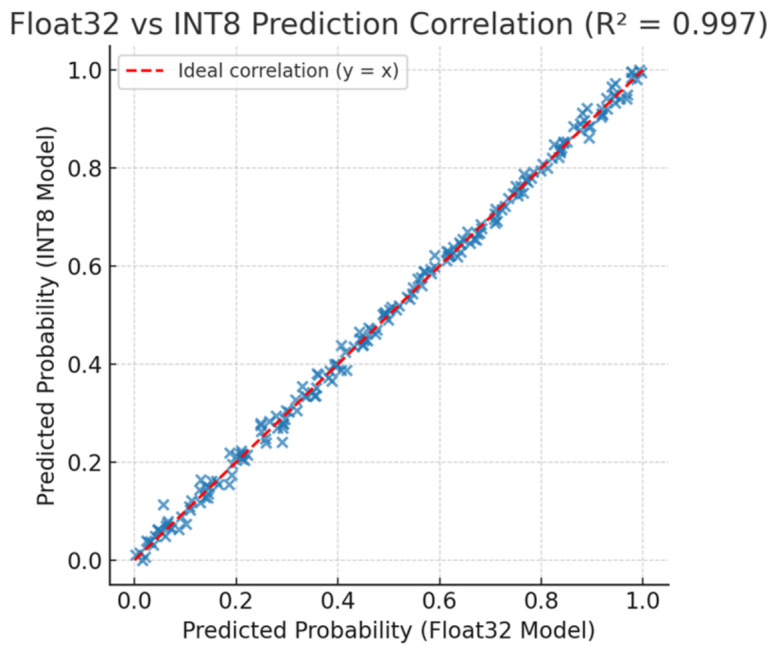
Scatter plot comparing predicted probabilities between Float32 and INT8 models. The strong linear correlation ($R^{2}=0.992$) confirms that quantization minimally affected the model’s predictive behavior.

Despite the considerable compression, the quantized model preserved more than 99% of the original performance, confirming the robustness of the learned representations. Figure 6 illustrates the correlation between floating-point and quantized model predictions on the validation set, showing a near-perfect linear relationship ($R^{2}=0.992$), which demonstrates that quantization did not significantly distort the decision boundaries.

This strong linear correlation ($R^{2}=0.992$) serves as statistical evidence that the INT8 quantization process preserved the decision boundaries established during training. Unlike pruning methods that remove neural connections, quantization retained the model’s structural integrity, allowing the decision logic learned in high-precision floating-point arithmetic to be faithfully executed on the integer-only arithmetic logic unit (ALU) of the ATmega328P (Microchip Technology Inc., Chandler, AZ, USA) with negligible accuracy degradation (<0.7%).

The inference benchmark on the Arduino UNO showed an average processing time of 200 ms per heartbeat, satisfying the requirements for real-time analysis of ECG signals sampled at 360 Hz. Memory profiling indicated that the quantized model occupied approximately 21.8 KB of Flash and 1.7 KB of SRAM, leaving sufficient space for signal acquisition, preprocessing, and display tasks.

This optimization validates the technical feasibility of deploying neural models for cardiac arrhythmia detection on 8-bit microcontrollers, eliminating the dependency on high-end ARM or GPU-based devices. In comparison with recent works using Raspberry Pi or STM32 platforms [33,34,36], the proposed approach achieves similar classification accuracy with over 90% lower hardware cost, strengthening its suitability for educational, experimental, and rural healthcare applications.

Overall, the quantization stage achieved a substantial improvement in computational efficiency with minimal accuracy degradation, establishing the foundation for full TinyML integration on ultra-low-cost embedded systems.

### 3.3. Real-Time Hardware Performance

The proposed system was physically implemented using an Arduino UNO (ATmega328P) microcontroller (Via Andrea Appiani, Monza, Italy), an AD8232 analog front-end module for ECG acquisition, and a 0.96-inch OLED display (SSD1306 controller) for visualization. The objective of this stage was to experimentally validate the real-time operation of the quantized 1D-CNN model within the memory and timing constraints of an 8-bit microcontroller.

The quantized model, integrated as a C array (model.h), executed entirely within the available 32 KB Flash memory and 2 KB SRAM of the Arduino UNO. Memory profiling indicated that the model and inference buffers occupied approximately 21.8 KB of Flash and 1.7 KB of SRAM, leaving a small but sufficient margin for signal acquisition, filtering, and display routines.

During testing, ECG signals were continuously acquired through the AD8232 sensor (Analog Devices, Norwood, MA, USA) at 360 Hz, emulating the sampling rate of the MIT-BIH dataset. The average inference time per heartbeat segment (250 samples) was measured using the Arduino’s internal timer, resulting in an average delay of 200 ms per beat. This measurement corresponds to the execution time of the neural network model only. Additional processing stages, such as signal filtering and R-peak detection, were executed separately and were not included in this latency measurement. Despite this, the system maintains real-time capability for beat-by-beat arrhythmia classification. Table 6 summarizes the main performance parameters obtained in the embedded implementation.

The reported current consumption corresponds to an average operating condition measured during continuous system execution.

**Figure 7:**
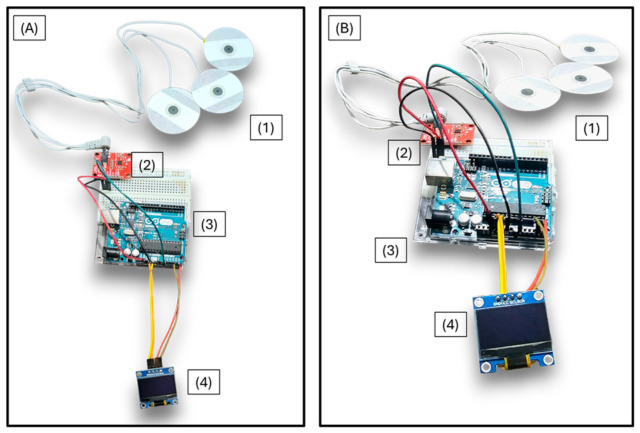
Photograph of the complete prototype illustrating the system’s components: (**A**) Front view of the prototype; (**B**) alternative view of the same configuration from a different perspective. The system includes: electrodes for signal acquisition (1), the AD8232 front-end module (2), the Arduino UNO microcontroller for processing (3), and the OLED screen for displaying the output (4).

Figure 7 shows the complete prototype configuration, including the electrodes for signal acquisition (1), the AD8232 analog front-end (2), the Arduino UNO microcontroller (3), and the OLED screen (4). The integration of these components demonstrates the feasibility of deploying a functional ECG classification system entirely on low-cost hardware. The device operates autonomously, without the need for external computing resources or network connectivity.

This figure illustrates the physical arrangement of the system components used during prototype-level testing under controlled laboratory conditions.

**Figure 8:**
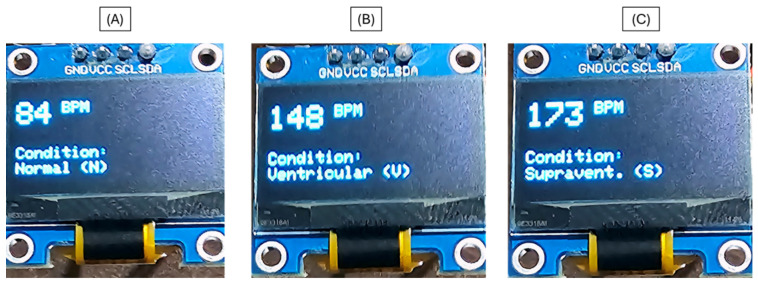
Real-time OLED display output of the TinyML-based arrhythmia classifier. The system shows heart rate (BPM) and the corresponding cardiac condition: (**A**) Normal (N), (**B**) Ventricular (V), and (**C**) Supraventricular (S). This interface provides immediate visual feedback, enabling standalone operation suitable for low-resource and rural healthcare environments.

Once the model was successfully deployed, the OLED interface provided a minimalistic and immediate visualization of the classification results. Each detected heartbeat was displayed in real time along with the calculated heart rate (BPM) and the corresponding condition label. Three representative cases are illustrated in Figure 8: (A) a Normal (N) rhythm at 84 BPM, (B) a Ventricular (V) arrhythmia at 148 BPM, and (C) a Supraventricular (S) arrhythmia at 173 BPM. This visualization confirms the correct operation of the quantized 1D-CNN model under strict memory and processing limitations, demonstrating the system’s capacity for on-device, interpretable inference in real time.

The system demonstrated stable operation during continuous monitoring sessions exceeding 30 min, without buffer overflow or memory fragmentation issues. Despite the limited processing capacity of the ATmega328P, the quantized model maintained consistent response times, validating the efficiency of the TinyML deployment pipeline. The use of the AD8232 analog front-end enabled practical ECG signal acquisition suitable for prototype-level validation under controlled conditions.

Moreover, the low-power operation was estimated based on the system’s current consumption during runtime. The device operates at a supply voltage of 5 V, and the average current consumption (approximately 40 mA) was measured using a digital multimeter connected in series with the power source. Based on the relation P = V × I, this results in an estimated power consumption of approximately 200 mW under typical operating conditions. It should be noted that this value represents an average estimate and does not include detailed profiling of transient or peak power consumption.

Overall, the results confirm that the combination of a quantized neural model and low-cost hardware provides a technically viable and affordable solution for real-time arrhythmia monitoring, supporting the main objective of this study—to demonstrate the feasibility of a TinyML-based embedded classification system for rural clinics in Mexico and other developing regions.

The real-time experiments were conducted using the prototype system with ECG signals acquired from 35 healthy volunteers under controlled, non-clinical conditions. These recordings were performed exclusively for functional validation of signal acquisition, filtering, heartbeat segmentation, OLED visualization, and real-time inference on the embedded platform.

It is important to emphasize that no annotated ground truth was available for these recordings, and therefore no classification performance metrics (e.g., accuracy, sensitivity, or F1-score) were computed from the real-time experiments. As a result, these tests do not constitute a quantitative evaluation of the model under real-world conditions.

Accordingly, the real-time experiments should be interpreted strictly as a validation of hardware operability and embedded inference feasibility, rather than as a clinical or diagnostic performance assessment.

Consequently, the prototype-level experiments should be interpreted strictly as a validation of hardware operability and embedded inference feasibility, rather than as a clinical evaluation of the classification model. These recordings were performed exclusively for functional validation of signal acquisition, filtering, heartbeat segmentation, OLED visualization, and real-time inference on the embedded platform.

Participation in the prototype-level tests was voluntary, and the participants were informed in advance about the technical purpose of the recordings. The experiments were non-invasive and limited to short-duration signal acquisition for hardware and algorithm verification purposes only. No clinical diagnosis, patient evaluation, or annotated dataset construction was performed during this stage. No personally identifiable clinical information was collected, and the recordings were used exclusively for internal technical validation of the prototype.

Therefore, the real-time prototype experiments should be interpreted strictly as a validation of hardware operability and embedded inference feasibility, rather than as a clinical or statistical evaluation of the classification model. In particular, these tests do not constitute evidence of model generalization under real-world low-cost acquisition conditions.

All prototype-level acquisitions were carried out for research-oriented technical validation only, under non-clinical conditions, and should not be interpreted as medical-grade ECG assessment.

### 3.4. Comparative Analysis with Related Works

Several studies have investigated automatic arrhythmia classification using the MIT-BIH Arrhythmia Database, employing a wide range of machine learning and deep learning architectures. Table 7 summarizes representative works published between 2020 and 2025, highlighting their model architectures, classification performance, and implementation characteristics. While most studies have achieved remarkable accuracy, only a few have demonstrated the feasibility of real-time inference on constrained embedded hardware.

Table 7 summarizes representative state-of-the-art approaches for arrhythmia classification using the MIT-BIH database, highlighting differences in model architecture, classification task, and deployment platform. It can be observed that most existing studies focus on multi-class classification problems, typically involving five or more heartbeat categories following the AAMI standard, and rely on deep learning architectures such as CNN, CNN–LSTM, or attention-based hybrid models. These approaches achieve very high accuracy values, often exceeding 98%, benefiting from large datasets and high-performance computing environments (e.g., GPUs or embedded processors with greater computational capabilities).

In contrast, the proposed method addresses a reduced three-class classification problem (Normal, Ventricular, and Supraventricular), which was intentionally selected to enable deployment on an ultra-low-cost 8-bit microcontroller. Additionally, while most compared works are implemented on desktop, GPU-based systems, or advanced embedded platforms, the proposed system operates entirely on an Arduino UNO without floating-point support. This represents a significantly more constrained hardware scenario in terms of memory, processing power, and energy consumption.

Therefore, although the proposed model does not surpass the highest accuracy values reported in the literature, it achieves competitive performance (97.6%) while drastically reducing hardware complexity and cost. This trade-off between accuracy and deployability is a central contribution of this work, demonstrating that real-time arrhythmia classification is feasible even under extreme resource constraints. Consequently, the comparison should be interpreted in the context of different problem formulations and deployment objectives, rather than as a direct one-to-one performance competition.

Deep hybrid architectures such as CNN–LSTM or CNN–BiLSTM–Attention combinations have achieved accuracies above 99% on the MIT-BIH dataset, benefiting from the use of powerful processors and large memory resources. For instance, Alamatsaz et al. [32] reported 98.24% accuracy using a CNN–LSTM hybrid model, while Najia and Faouzi [36] proposed an enhanced hybrid framework combining CNN, BiLSTM, and attention mechanisms that achieved 99.20% accuracy on ECG segment classification.

Similarly, Hua et al. [34] developed a one-dimensional CNN architecture that reached 99.24% accuracy for five arrhythmia classes, demonstrating the potential of deep convolutional models when deployed on high-performance computing environments. Although these approaches exhibit outstanding classification capabilities, they are primarily executed on desktop or server hardware, which limits their practical applicability in embedded or portable systems.

On the other hand, Kim et al. [31] presented a TinyML-based CNN (TinyCES) for arrhythmia monitoring on an Arduino platform, achieving approximately 97% accuracy. This work confirmed the feasibility of performing real-time ECG classification directly on low-cost microcontrollers.

More recently, Busia et al. [37] introduced a Tiny Transformer architecture for ultra-low-power arrhythmia detection, demonstrating nearly 99% accuracy on GAP9 microcontrollers, thereby extending TinyML capabilities to transformer-based models. Likewise, Sun et al. [33] proposed a CNN–LSTM–SE model with channel attention that achieved 98.5% accuracy, although the implementation still relied on GPU-based hardware. Additionally, Eleyan et al. [27] proposed a mobile deep learning-based ECG device (RHYTHMI) for heart disease prediction, highlighting the growing trend toward portable and accessible diagnostic systems. In a related work, Eleyan et al. [28] introduced a spectrogram-based arrhythmia classification approach using a multi-channel deep learning model with feature fusion, achieving high classification performance through time–frequency representations. These contributions further support the relevance of combining efficient signal representations with deployable machine learning models in practical healthcare scenarios.

It should be noted that many of the compared studies address a larger number of arrhythmia classes (typically five or more), which inherently increases classification complexity and clinical scope. In contrast, the present work focuses on a reduced three-class problem to enable deployment on ultra-low-cost hardware. Therefore, the comparison should be interpreted in the context of a deliberate trade-off between classification scope and embedded feasibility. While this simplification supports TinyML deployment, it also limits the clinical completeness of the proposed system.

A comparison with state-of-the-art architectures (Table 7) reveals a clear trade-off between computational efficiency and absolute accuracy. While deep learning models utilizing BiLSTM, attention mechanisms, or transformer-based architectures often achieve accuracies exceeding 99%, these solutions typically require substantially greater memory, processing power, and hardware complexity, making them less suitable for deployment on ultra-low-cost standalone devices. In contrast, the present work intentionally adopts a compact and shallow 1D-CNN architecture to prioritize real-time embedded inference, low memory footprint, and minimal energy consumption. Therefore, the contribution of this study lies not in architectural novelty, but in demonstrating that a standard and lightweight 1D-CNN can be successfully adapted, quantized, and deployed on ultra-low-cost embedded hardware. This engineering-oriented contribution highlights the feasibility of real-time inference under extreme memory and computational constraints, which remains a key challenge in TinyML applications.

### 3.5. Discussion and Feasibility

The results confirm that efficient arrhythmia classification is achievable on low-cost hardware through proper quantization and embedded optimization. It is important to emphasize that the main contribution of this study is centered on engineering feasibility rather than algorithmic innovation. In particular, the work demonstrates that a standard deep learning model can be effectively adapted for execution on an 8-bit microcontroller, where constraints on memory, processing power, and energy consumption are significantly more restrictive than in conventional machine learning environments. Specifically, the work demonstrates that a lightweight and well-established 1D-CNN can be successfully adapted to the TinyML paradigm and executed on an 8-bit Arduino UNO while preserving competitive classification performance. Beyond technical feasibility, the system’s practical relevance lies in its high Sensitivity (97.6%) under benchmark evaluation conditions for detecting ventricular and supraventricular beats.

From a practical perspective, the proposed system should be interpreted as a technological proof-of-concept rather than a clinically validated diagnostic device. Its primary value lies in demonstrating that meaningful ECG classification can be achieved on ultra-low-cost hardware using the TinyML paradigm.

While the current implementation presents limitations in terms of signal quality and clinical validation, it establishes a foundation for the development of more robust and scalable systems. In particular, future implementations on more capable microcontrollers (e.g., ARM Cortex-M or ESP32 platforms) could significantly improve performance, connectivity, and integration into real-world healthcare workflows.

Therefore, the practical significance of this work resides in bridging the gap between advanced machine learning models and their deployment in constrained environments, rather than in delivering a finalized medical product.

It is important to emphasize that the selection of the Arduino UNO platform does not represent an optimal hardware choice for ECG acquisition or processing systems. More advanced platforms, such as ARM Cortex-M-based microcontrollers (e.g., STM32) or embedded systems like Raspberry Pi, provide significantly higher computational performance, hardware floating-point support, and improved energy efficiency. It is important to note that the AD8232-based acquisition system does not provide clinical-grade ECG quality. The signal quality is influenced by multiple factors, including electrode placement, motion artifacts, and skin–electrode impedance. Therefore, the hardware implementation should be interpreted as a low-cost, proof-of-concept solution rather than a fully optimized biomedical instrumentation system.

However, the objective of this work was to explore the minimal feasibility boundary of TinyML deployment under extreme resource constraints. In this sense, the Arduino UNO serves as a representative baseline of highly constrained hardware, allowing us to evaluate whether real-time arrhythmia classification is achievable without floating-point operations and with very limited memory resources. Therefore, the proposed system should be interpreted as a proof-of-concept lower-bound implementation rather than as an optimized engineering solution for real-world medical devices.

Nevertheless, this study has limitations inherent to its experimental design. First, the model was trained and quantitatively validated exclusively on the MIT-BIH Arrhythmia Database. Although this dataset is considered a gold-standard benchmark, it was collected under controlled clinical conditions and may not fully capture the noise profiles, motion artifacts, and signal variability associated with low-cost acquisition systems used in field settings. Second, the dataset partitioning was performed at the heartbeat level (beat-wise), without enforcing inter-patient separation. As a result, samples from the same patient may appear in both training and testing subsets, introducing data leakage. This can lead to optimistic performance estimates, as the model may learn patient-specific morphological patterns rather than fully generalizable features. Consequently, the reported results should not be interpreted as a strict patient-independent evaluation. Accordingly, the reliability of the present results must be understood within the scope of a technical feasibility study. The current evaluation demonstrates that the proposed lightweight model can achieve strong benchmark performance and be executed on an ultra-constrained microcontroller, but it does not establish patient-independent clinical reliability. Future work should therefore incorporate patient-wise partitioning and external validation datasets to determine whether the model generalizes under more realistic clinical conditions. Therefore, the reported metrics should be interpreted as a technical validation of the embedded system rather than a fully patient-independent evaluation. Finally, the classification task was reduced from the full AAMI 5-class scheme to a 3-class problem, which reduces clinical granularity and excludes other relevant heartbeat categories. This simplification enables deployment on ultra-constrained hardware, but it also limits the direct applicability of the system as a comprehensive clinical arrhythmia classifier.

As a result, the current study does not establish real-world generalization of the classifier under practical deployment conditions. Instead, it demonstrates benchmark-level model performance together with prototype-level embedded feasibility.

Additionally, the real-time testing performed in this study was limited to prototype-level validation using signals acquired from healthy volunteers under controlled conditions. These recordings were not annotated by clinical experts and therefore do not include ground truth labels for arrhythmia classification.

As a result, it is not possible to assess the real-world diagnostic performance of the proposed system based on these experiments. The real-time tests were designed exclusively to verify the correct operation of signal acquisition, preprocessing, segmentation, and embedded inference on the microcontroller.

Therefore, a clear gap remains between benchmark-based model evaluation (using the MIT-BIH dataset) and real-world validation using low-cost acquisition hardware. This limitation implies that the reported classification performance cannot be directly extrapolated to practical deployment scenarios. Future work must include annotated datasets acquired under real-world conditions in order to evaluate robustness against noise, motion artifacts, and sensor variability.

The reported latency corresponds to model inference only; therefore, future work should consider full end-to-end latency optimization, including preprocessing stages, to further improve system responsiveness. A more detailed power characterization, including peak and idle consumption analysis, is left as future work.

It is also important to clarify that the present work does not aim to outperform state-of-the-art deep learning architectures in terms of absolute accuracy. Instead, the primary contribution lies in demonstrating that a standard and relatively shallow 1D-CNN model can be effectively adapted, quantized, and deployed on an 8-bit microcontroller while maintaining competitive performance. This engineering-oriented perspective differentiates the work from purely algorithmic studies.

While hybrid architectures such as CNN–LSTM or CNN–GRU have demonstrated superior performance in arrhythmia classification tasks, their computational complexity and memory requirements make them unsuitable for deployment on ultra-constrained 8-bit microcontrollers such as the Arduino UNO. In this study, the selection of a compact 1D-CNN architecture was a deliberate design decision to ensure real-time execution within strict hardware limitations. Future work will explore the implementation of more advanced hybrid architectures on higher-performance embedded platforms (e.g., ARM Cortex-M or ESP32), where increased computational capacity may allow improved classification performance without compromising real-time operation.

Accordingly, the present study should be interpreted as a benchmark-guided embedded implementation study, where the quantitative evaluation and the prototype-level validation play complementary but distinct roles.

From a clinical perspective, the analysis of misclassification errors is particularly important. In this study, false negatives in pathological classes (ventricular and supraventricular beats) represent the most critical limitation, as they correspond to missed abnormal events. Although the model achieved high sensitivity values, a detailed risk-based evaluation of these errors was not performed. Therefore, the current results should be interpreted as technical performance indicators rather than as a comprehensive clinical reliability assessment.

Future implementations of the proposed system could benefit from migrating to more capable platforms such as STM32, ESP32, or other ARM-based microcontrollers, which would allow more complex models, improved signal processing pipelines, lower latency, and enhanced energy efficiency. These platforms represent a more suitable choice for real-world deployment scenarios. Future work should incorporate patient-wise partitioning strategies to provide a more clinically realistic evaluation of model generalization.

## 4. Conclusions

This work presented the design, implementation, and validation of a real-time cardiac arrhythmia classifier based on the TinyML paradigm, optimized for deployment on ultra-low-cost embedded hardware. The study does not propose a novel deep learning architecture; instead, its main contribution lies in demonstrating that a compact and well-established 1D-CNN can be successfully adapted, quantized, and deployed on an ultra-low-cost 8-bit microcontroller while preserving real-time performance and competitive classification accuracy under benchmark conditions.

The proposed model achieved an accuracy of 97.6% after quantization, with an inference time of approximately 200 ms per heartbeat and total memory usage of less than 24 KB, enabling true real-time, beat-by-beat classification. The prototype, which combines the AD8232 analog front-end and a 0.96-inch OLED display, successfully executed all signal acquisition, processing, and visualization tasks locally—without the need for external computing resources or network connectivity. This confirms the technical feasibility of implementing autonomous diagnostic tools on 8-bit microcontrollers.

Compared with existing approaches in the literature, the proposed system maintains competitive accuracy while drastically reducing hardware complexity, energy consumption, and cost (under 25 USD). These characteristics make it particularly suitable for educational purposes, wearable applications, and medical support in rural or resource-limited settings where specialized cardiology services are often unavailable.

The results validate the potential of TinyML as an enabling technology for accessible and decentralized healthcare solutions. Future work will focus on extending the classifier to support additional arrhythmia categories, improving robustness against real-world noise, and migrating the architecture to higher-performance microcontrollers (such as STM32 or ESP32) to enable wireless connectivity and long-term patient monitoring. Ultimately, this research establishes a technological foundation for the development of affordable, intelligent medical instruments capable of supporting future development of low-cost cardiac monitoring technologies and future decision-support workflows in rural clinics across Mexico, contributing to equitable healthcare access and preventive cardiology in regions with limited medical infrastructure. In particular, the present implementation is limited to three heartbeat classes, and therefore does not cover the full spectrum of arrhythmia categories defined by the AAMI framework. This should be considered when interpreting its potential clinical applicability.

It is important to highlight that the proposed system is not intended for direct clinical diagnosis, but rather as a proof-of-concept platform demonstrating the feasibility of embedded arrhythmia classification. Its potential practical impact lies in enabling low-cost screening tools and supporting future developments toward accessible healthcare technologies in underserved regions.

However, because the quantitative validation was performed exclusively on the MIT-BIH benchmark, the present results should not be interpreted as evidence of generalization to noisy real-world ECG signals acquired from low-cost hardware.

Nevertheless, the current system should be considered a proof-of-concept prototype, and further validation using annotated ECG signals acquired directly from the proposed hardware, as well as patient-based clinical studies, is required before real-world medical deployment. Additionally, the absence of inter-patient data separation may lead to optimistic performance estimates, and should be addressed in future validation studies. Additionally, the current system does not include explainability mechanisms, which are essential for clinical trust. Future work will focus on incorporating lightweight interpretability techniques to improve transparency and facilitate adoption in medical contexts.

Additionally, future work should include a more detailed clinical evaluation of misclassification errors, particularly focusing on false negatives and class-wise specificity, to better assess the system’s reliability in real-world screening scenarios.

## Figures and Tables

**Table 1:** Hyperparameters and configuration of the proposed 1D-CNN model.

Layer	Type	Filters/Units	Kernel Size	Activation	Output Shape
1	Conv1D	16	3	ReLU	(248, 16)
2	MaxPooling1D	–	2	–	(124, 16)
3	Conv1D	32	3	ReLU	(122, 32)
4	Flatten	–	–	–	3904
5	Dense	64	–	ReLU	64
6	Dense (Output)	3	–	Softmax	3 Classes

**Table 2:** Training hyperparameters of the 1D-CNN model.

Parameter	Value
Learning rate	0.001
Batch size	32
Epochs	100
Optimizer	Adam
Loss function	Categorical Cross-Entropy
Early stopping	Patience = 10 epochs

**Table 3:** Connection table of the electronic system components.

Component	Module Pin	Arduino UNO Pin	Description
AD8232	3.3 V/5 V	5 V	Battery-powered supply during experiments
	GND	GND	Common ground
	OUT	A0	ECG analog output
	LO+	11 (optional)	Lead-off detection
	LO−	10 (optional)	Lead-off detection
	SDN	–	Not connected (active by default)
OLED 0.96″ (SSD1306 $I^{2}$C)	VCC	5 V	Battery-powered supply during experiments
	GND	GND	Common ground
	SDA	A4	$I^{2}$C data line
	SCL	A5	$I^{2}$C clock line

**Table 4:** Average cross-validation results over five folds.

Metric	Mean (±SD)
Accuracy	98.27 ± 0.41%
Precision	97.92 ± 0.55%
Recall (Sensitivity)	97.60 ± 0.73%
F1-Score	97.76 ± 0.61%
AUC (Area under ROC curve)	0.993 ± 0.002

**Table 5:** Comparison between the floating-point and quantized versions of the proposed 1D-CNN model.

Model Version	Size (KB)	Accuracy (%)	Memory Reduction (%)	ΔAccuracy
Float32 (original)	85.2	98.3	—	—
INT8 (TinyML)	21.9	97.6	74.3	−0.7

**Table 6:** Summary of embedded system performance for real-time arrhythmia classification.

Parameter	Value
Sampling Frequency	360 Hz
Inference Time per Beat	200 ms
Flash Memory Usage	21.8 KB
SRAM Usage	1.7 KB
Benchmark Classification Accuracy (INT8 model)	97.6%
Power Supply Voltage	5 V
Average Current Consumption	40 mA
Total System Cost	<25 USD

**Table 7:** Comparison of representative arrhythmia classification studies using the MIT-BIH database.

Study	Model/Architecture	Classes/Task	Accuracy (%)	Deployment/Hardware
Kim et al. (2023) [31]	CNN (TinyML prototype)	Binary/Multiclass ECG	∼97	Arduino-based TinyML system
Alamatsaz et al. (2022) [32]	Lightweight hybrid CNN–LSTM	8 arrhythmia types + normal	98.24	General-purpose computing (non-MCU)
Sun et al. (2024) [33]	CNN–LSTM–SE with channel attention	5 classes (N, A, V, L, R)	98.5	CPU/GPU environment
Hua et al. (2020) [34]	1D CNN (end-to-end)	5 AAMI classes	99.24	Server/desktop
Najia & Faouzi (2025) [36]	CNN + BiLSTM + Attention	5 AAMI classes (N, S, V, F, Q)	99.20	Research setup, non-embedded
Busia et al. (2025) [37]	Tiny transformer architecture	5 arrhythmia classes	98.97	GAP9 ultra-low-power processor
Eleyan et al. (2024) [27]	Mobile DL-based ECG system (RHYTHMI)	3 classes (NSR, CHF, ARR)/heart disease prediction	98.52	Mobile/portable device
Eleyan et al. (2024) [28]	Spectrogram + multi-channel DL (feature fusion)	3 classes (MIT-BIH + BIDMC) and 5 classes (MIT-BIH)	99.80 (3-class); 99.76 (5-class)	GPU/non-embedded
This Work (2025)	1D-CNN (TinyML)	3 classes (N, V, S)	97.6	Arduino UNO (8-bit MCU, standalone)

## Data Availability

Publicly available datasets were analyzed in this study. The MIT-BIH Arrhythmia Database can be found on PhysioNet: https://doi.org/10.13026/C2F305.
